# An Innovative Tube Hydro-Joining Process Combining Piercing, Hole Flanging and Nut Inlaying

**DOI:** 10.3390/ma18091990

**Published:** 2025-04-28

**Authors:** Yeong-Maw Hwang, Hong-Nhan Pham, Ze-Wei Ho, Yu-Jen Wang

**Affiliations:** Department of Mechanical and Electro-Mechanical Engineering, National Sun Yat-sen University, No. 70, Lien-Hai Rd., Kaohsiung 804, Taiwan; nhanck26@gmail.com (H.-N.P.); m103020065@g-mail.nsysu.edu.tw (Z.-W.H.); yjwang@mail.nsysu.edu.tw (Y.-J.W.)

**Keywords:** aluminum alloy tube, finite element analysis, hydro-joining, hydro-piercing, nuts inlaying, punch shape design, tube hydroforming

## Abstract

This paper proposes a novel tube hydro-joining process, which combines piercing, hole flanging, and nut inlaying. The nut punch shape design proposed by this paper can deliver three advantages of no scrap, no oil leakage, and longer flange length, which can achieve stronger clamping force and accordingly increase the pull out load. First, we use the finite element analysis to investigate the elasto-plastic deformation of the aluminum alloy A6063 tube during the hydro-joining process. A punch-shaped nut with a tapered locking part is designed to increase the elasto-binding strength of the pierced tube and the pull out load of the inlayed nut. The effects of hydro-joining loading paths on the formability of the A6063 tubes and punch-shaped nuts are examined. Additionally, the effects of fit zone size, nut punch stroke length, internal pressure, nut diameter, and the die hole diameter on the pull out load and twisting torque are explored. Finally, experiments on hydro-joining of A6063 tubes are conducted to validate the finite element modeling and the simulation results.

## 1. Introduction

Energy saving and carbon dioxide reduction are critical global concerns in aerospace and transportation. Tube hydroforming (THF) processes are widely used in manufacturing lightweight parts for bicycles, automobiles, and aerospace applications. Compared with conventional metal forming processes, THF processes reduce workpiece and tool costs and product weight. THF processes also improve structural stability, strength, and stiffness in parts such as front and rear axles, exhaust system components, and body frames [1,2]. Recent developments in THF have incorporated piercing, flanging, or joining processes to create hydropiercing, hydroflanging, and hydro-joining, which are more efficient than a single process and reduce the total weight of the final product [3,4].

Studies investigating the hydropiercing and hydroflanging of sheets or tubes have yielded valuable insights into these processes. For example, Fracz et al. [5] investigated how punch geometry affects sheet thickness distribution during the hole-flanging process. Their study used cylindrical (flat-bottomed), hemispherical, and conical punch geometries in experiments and numerical simulations and compared the results. Additionally, Kacem et al. [6] used a conical punch to characterize and numerically predict material failure for two aluminum sheets during the hole-flanging process. Their study developed a fracture criterion on the basis of local tension strain measures and compared numerical strain predictions with the experimental outcomes.

The composite process of hydroforming, punching, and inlaying can be used to efficiently join tubular materials with other components to form structural parts. Thipprakmas et al. [7] proposed a fine-blanked-hole-flanging process to investigate sheet deformation using the finite element (FE) simulations. The flanged shapes from the simulations closely matched the experimental results. The results indicated that the fine-blanked-hole-flanging process produced superior flanged shapes and improved flangeability compared with hole flanges produced using the conventional-hole-flanging process. Smith [8] described the hydropiercing process, which typically involves using a punch to penetrate the workpiece surface and utilizing internal liquid pressure to push the material against the punch and the die cavity. This method is primarily suitable for producing large holes. Moreover, Wu et al. [9] employed six ductile fracture theories to predict fracture initiation and propagation. Subsequently, they compared the simulation results and experimental outcomes, identifying the theories that could predict fracture propagation and shear planes. Furthermore, Qian et al. [10] investigated the edge stretchability of predamaged holes caused by punching. Their research incorporated initial surface irregularities from punching into the flanging process, demonstrating the necessity of assessing preexisting damage at the hole edges before punching. Hwang et al. [11] designed three punch shapes to study the thickness distribution of flanged tubes during hydroflanging and evaluate fluid leakage between the punch and the flanging areas. They concluded that sharper punches tend to cause failure near the tip region, whereas blunter punches cause failure near the annular region. Additionally, the tubes bulged at higher internal pressures, with increased thickness near the annular region and reduced thickness beneath the punch. Additionally, Liu et al. [12] examined the differences between three transition zones produced by piercing punches and flanging punches to explore the effects of punch shape on the geometric profile and formation quality of the holes. Mizumura et al. [3] studied methods for joining hydroformed components with other parts and developed a technique termed nut inlaying. They designed nine nut shapes and conducted evaluations on the basis of six criteria. Mizumura et al. [4] also developed hydroburring, a method that expands a hole made by hydropiercing while maintaining internal pressure. This method enables bolt joining by tapping screw threads into the hole or inlaying a nut. Choi et al. [13] analyzed the tube deformation behavior surrounding a hole produced by hydropiercing, investigating the association between the deformation radius and rollover under varying punch diameters and internal pressures. Hwang and Wu [14] proposed a compound forming process combining crushing, hydroforming, and calibration to hydroform rectangular aluminum alloy (A6061-O) tubes. Their process achieved a uniform thickness distribution, reduced internal pressure, and lower clamping force than traditional hydroforming. Hwang et al. [15] subsequently used FE simulations and experiments to investigate the effects of punch shape and parameters such as punch stroke length and internal pressures on the surface characteristics of pierced holes in SPFC590Y carbon steel tubes. Specifically, they examined deformation mechanisms that resulted in superior surface quality. Hwang and Tsai [16] introduced a THF process with a novel movable die and loading path design to manufacture irregular bellows with minimal thinning ratios. Two feeding methods were used to minimize thinning. The FE simulation software DEFORM 3D (V11.0.2) was used to analyze tube plastic deformation in the die cavity. The movable die design with an optimized die gap reduced the internal forming pressure required to one-sixth of the pressure required without the movable die.

Park et al. [17] developed an advanced sealing system to prevent fluid leakage during the hydroforming process. The system comprised a die spring, cylindrical sleeve, and a punch with an end fillet. Axial pressure between the punch and sleeve tightly sealed the tube end, increasing as the axial feed intensified. The system’s feasibility was experimentally verified by hydroforming a nonaxisymmetric part. Additionally, Yu et al. [18] introduced a quantitative method to evaluate the effects of crack size and material anisotropy on edge stretchability during sheet punching. The method used the effective failure strain ratio as an index. Numerical studies have investigated the interaction effect of cracks and anisotropy on the edge stretchability during hole stretching. The results reveal that cracks tended to form along the direction with the lowest r-value within the sheet plate. Experiments involving punching and hole expansion using dual-phase steel validated this conclusion. Finally, Kumar et al. [19] used FE simulations and experiments to investigate the effect of punch head profiles on the deformation behavior of AA5052 alloy sheets in stretch-flanging processes. Six punch geometries were tested. The results of the simulations and experiments revealed that the circumferential strain, radial strain, and punching load were lowest with a hemispherical punch profile.

Patne et al. [20] investigated the tightening torque of bolts joined with plastic components, highlighting problems such as thread failure or indentation when the material strength considerably exceeded the bolt strength, and vice versa. Zhang et al. [21] introduced movable sleeves in THF to enhance tube material flow, improve the uniformity of rounded corner thickness, and achieve more consistent thickness distribution with rounded corners with a smaller radius. This approach also reduced internal pressure and side-cylinder feeding requirements to address clamping defects. Additionally, Katsuya et al. [22] proposed a hydraulic impact method for drilling holes in aluminum pipes. This technique eliminated the requirement for punch alignment and enabled the creation of round or square holes on flat plates and pipes using dies alone. Finally, Manabe and Fuchizawa [23] reviewed the development history of THF technology, discussing hydroforming applications, hydroforming processes, and advances in lightweight structural designs. They also summarized the challenges and trends associated with this processing technique.

Sun et al. [24] explored the plastic deformation behavior of a square tube in square hole hydro-piercing processes experimentally and numerically. The effects of internal pressure and punch corner radius on the deformation sequence and the collapse behavior were investigated. They concluded that the collapse was related to the internal pressure but had little dependence on the punch corner radius. Uhe and Meschut [25] presented an approach to the advanced self-piercing riveting of ultra-high-strength steel 22MnB5 sheets. By combining experimental and numerical methods, a major improvement in rivet performance was achieved through a sweeping modification of the rivet material and the heat treatment. The new rivet operation can increase the joinability of 22MnB5 from a thickness of 1.7 mm to 2.3 mm.

**Figure 1:**
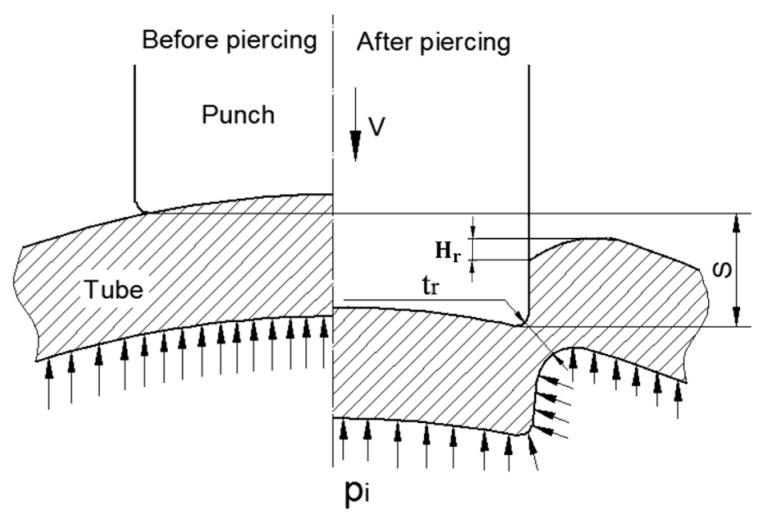
Contact configurations between punch and tube before and after hydro-piercing [15].

**Figure 2:**
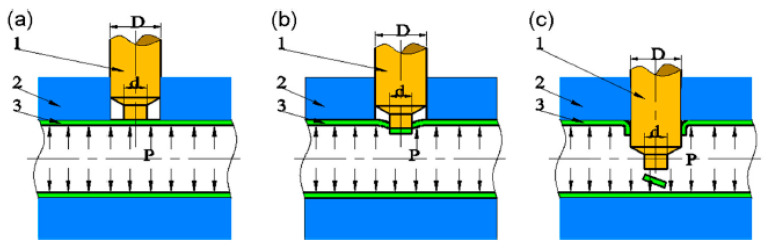
Process and principle of hydro-piercing-flanging (1 piercing-flanging punch, 2 die, 3 tube). (**a**) Hydroforming. (**b**) Hydro-piercing. (**c**) Hydro-flanging [10].

**Figure 3:**
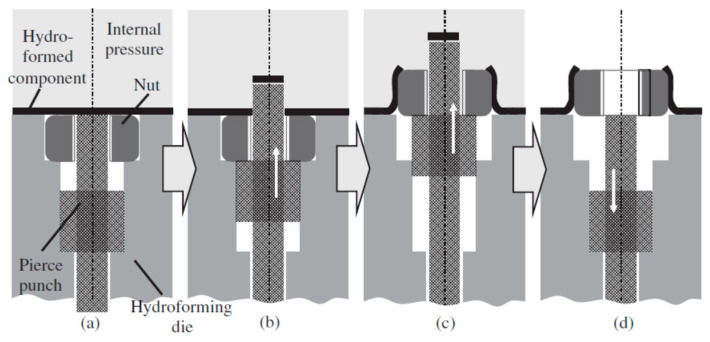
Process of nut inlaying into hydroformed component [3]. (**a**) Initial state; (**b**) Piercing; (**c**) Flanging and inlaying; (**d**) Punch withdrawing.

Tube hydro-joining involves hydro-piercing, hole flanging, and nut inlaying. Some papers [15,22,24] focused on hydro-piercing processes. For example, one of the present authors [15] proposed a punch shape design and investigated the effects of various parameters such as punch strokes, internal pressures, etc. on tube deformation and fracture mechanism in tube hydro-piercing processes of SPFC590Y carbon steel tubes, where its schematic is shown in Figure 1. Many researchers [3,4,5,6,7,10,11] focused on hole-flanging processes of sheets or tubes. For example, Liu et al. [10] proposed a punch shape design and investigated the effects of punch shape on geometrical profile and quality of pierced and flanged holes by experiments and simulations with three different shapes of transition zone between the piercing punch and the flanging punch. The geometric configurations between the punch, tube, and die are shown in Figure 2. Concerning the hydro-joining process combining piercing, hole flanging, and nut inlaying, Mizumura et al. [3] proposed a compound process combining hydro-burring and nut inlaying with a designed punch shape, as shown in Figure 3. However, with this method, the pierced scraps may result in blocking of the oil pipe and cause fluid flow problems. On the other hand, oil leakage around the punch surface may occur in the hydro-piercing and hydro-burring processes, which leads to the pressure drop of the internal pressure. In this paper, a novel hydro-joining processes combining piercing, flanging, and inlaying is proposed. The punch nut shape design proposed by this paper can deliver three advantages of no scrap, no oil leakage, and longer flange length, which can achieve stronger clamping force and accordingly increase the pull out load.

The present study investigated the parameters and loading paths influencing the joining conditions of aluminum alloy A6063-T4 tubes and punch-shaped nuts during hydro-joining. Using DEFORM 3D, this study simulated the hydroforming process of inlaying nuts into tubes. This study also assessed the effects of tapered locking part dimensions on the pull out load of the punch-shaped nut and validated the findings through experiments using a specially designed die set.

## 2. Finite Element Simulation Setup

### 2.1. Hydro-Piercing and Nut Inlaying

**Figure 4:**
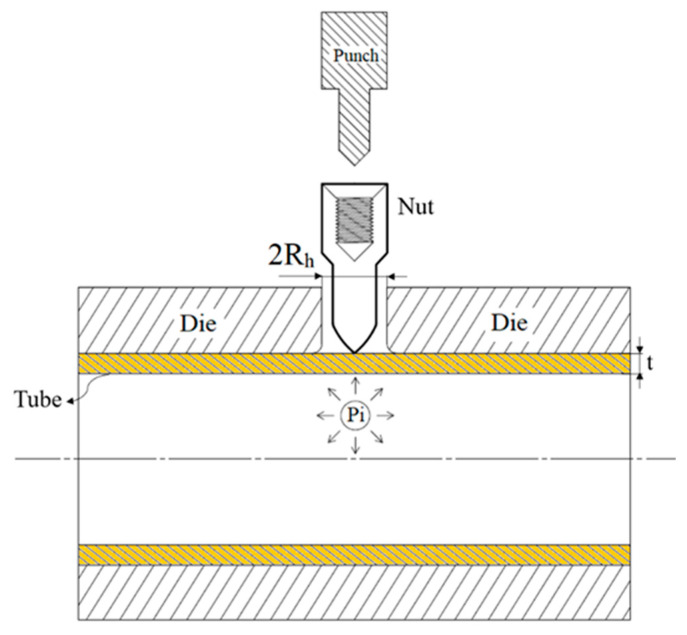
Initial positional relationship of the parts in the hydro-joining process.

This process expanded on the study of Hwang et al. [11]. The material used was aluminum alloy A6063, which was cut into test specimens and annealed before being subjected to tensile tests. The initial positional relationship of the parts, with the punch-shaped nut, tube, die, and punch, is illustrated in Figure 4.

**Figure 5:**
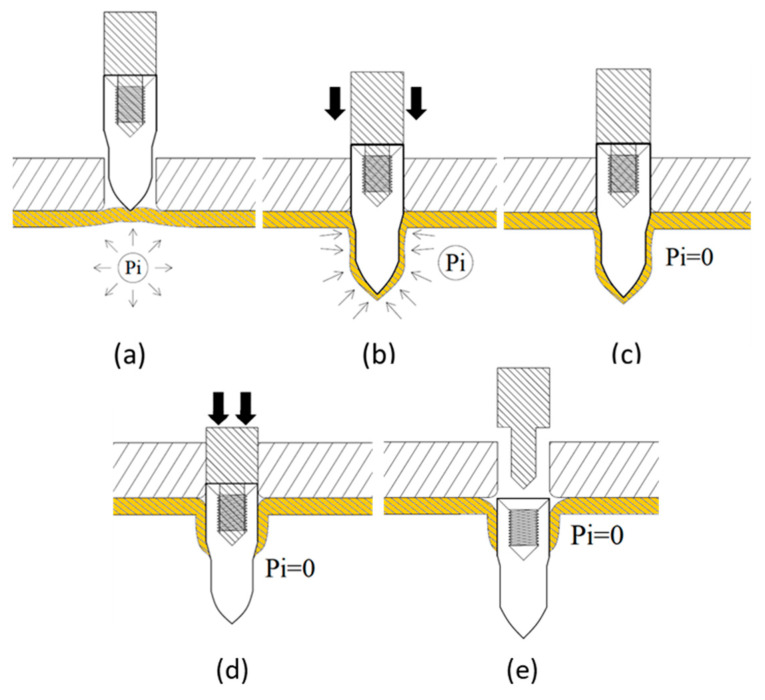
Schematic of the processing procedure. (**a**) Pressurizing; (**b**) Flanging; (**c**) Decompression; (**d**) Inlaying; (**e**) Punch withdrawing.

**Figure 6:**
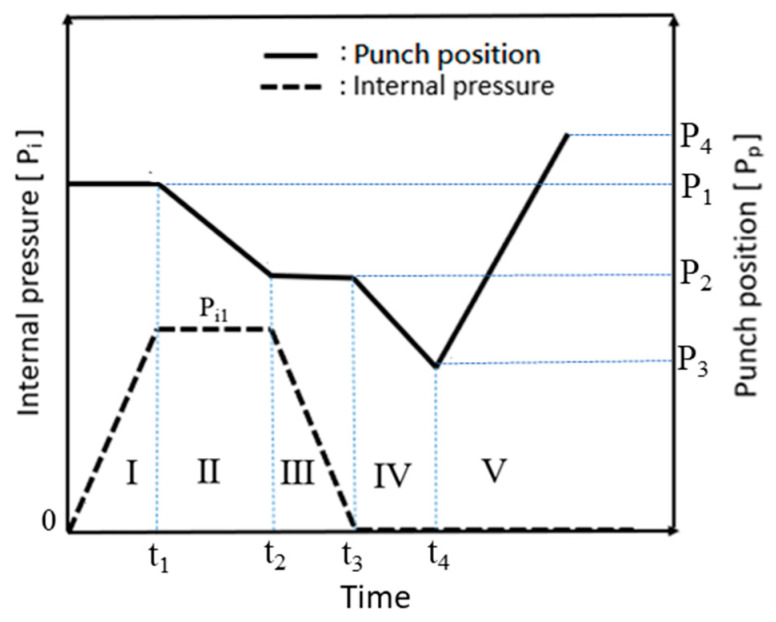
Diagram depicting internal pressure variations and punch paths.

The hydro-joining process comprised five stages (Figure 5 and Figure 6). In Figure 5a (Stage I), internal pressure was applied to expand the tube into the desired shape, establishing internal support. In Figure 5b (Stage II), the punch-shaped nut moved downward to punch and flange the aluminum alloy after reaching the specified internal pressure. Internal pressure and elastic tube recovery—resilience forces counteracted the downward force of the punch—prevented excessive indentation. The optimal point where the downward force was less than the tube’s support force was subsequently identified using material flow diagrams. In Figure 5c (Stage III), internal pressure was released to enable sufficient elastic tube material recovery during the inlaying stage and ensure adequate clamping force. Figure 5d depicts Stage IV, in which the punch continued to move downward, embedding the punch-shaped nut. At this stage, because the downward force was lower than the tube’s support force, further indentation did not occur. Finally, in Figure 5e (Stage V), the punch was retracted upward, leaving the nut in place. In tests conducted during this stage, the punch and nut were extracted together to determine the pull out load.

**Figure 7:**
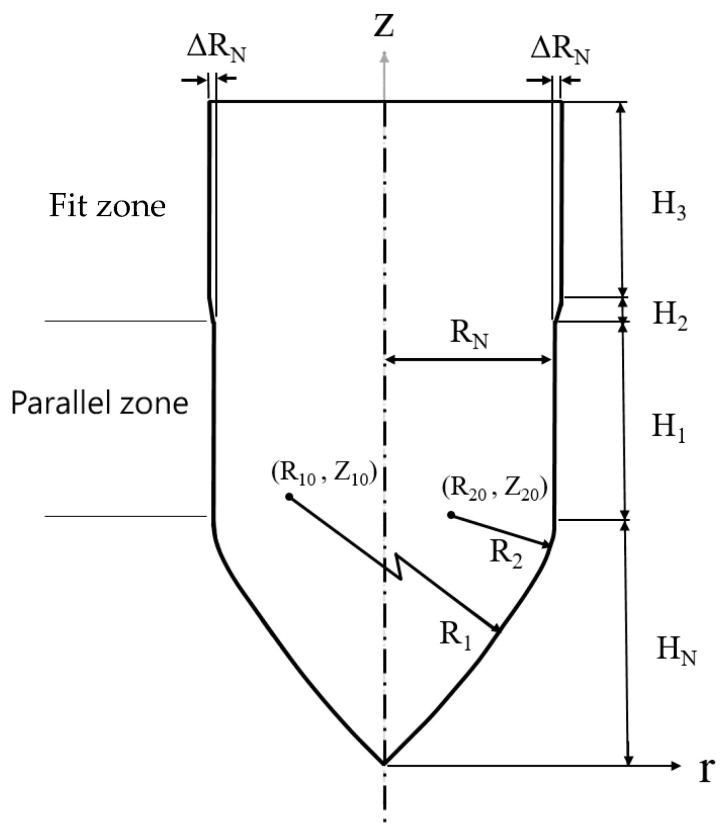
Nut design.

A detailed design of the punch-shaped nut is illustrated in Figure 7. The nut consisted of three zones: (1) Conical zone: The first area to contact the tube, the conical zone was responsible for punching. To prevent fluid leakage during the punching process, a circular arc with a radius (R_1_) of 20 mm was used for the top of the nut. (2) Parallel zone: Connected to the conical zone through an external rounded corner with a radius (R_2_) of 2 mm, this zone formed the parallel tube wall and has a diameter of 2R_N_ (6.9 mm) and a length of 4 mm. This design ensured sufficient contact area and pressure between the tube wall and the nut, preventing fluid leakage from the tube at high pressures. (3) Fit zone: The fit zone exhibited a gradually increasing radius relative to the parallel zone, with a diameter of 2R_N_. During the inlaying stage, in the absence of internal pressure, this zone compensated for the gap caused by the tube material’s elastic recovery after hydraulic pressure was released, enhancing the clamping force. Detailed nut design parameters are listed in Table 1.

The top side of the nut is used to connect the punch, as shown in Figure 5. The top side has no influence on the elasto-plastic deformation of the tube in the hydro-joining process. Accordingly, the top side of the nut was not shown in Figure 7.

### 2.2. Simulation Conditions

**Figure 8:**
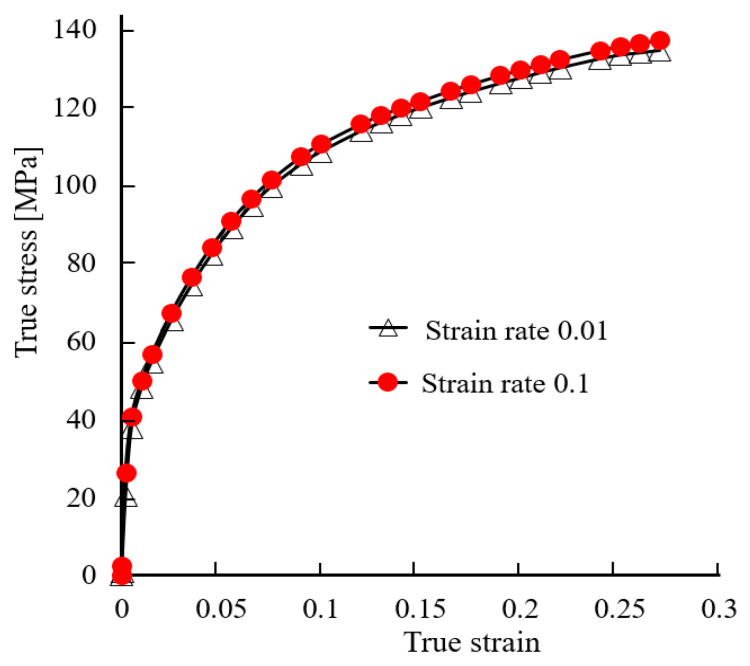
Stress-strain curves of aluminum alloy tube A6063.

**Figure 9:**
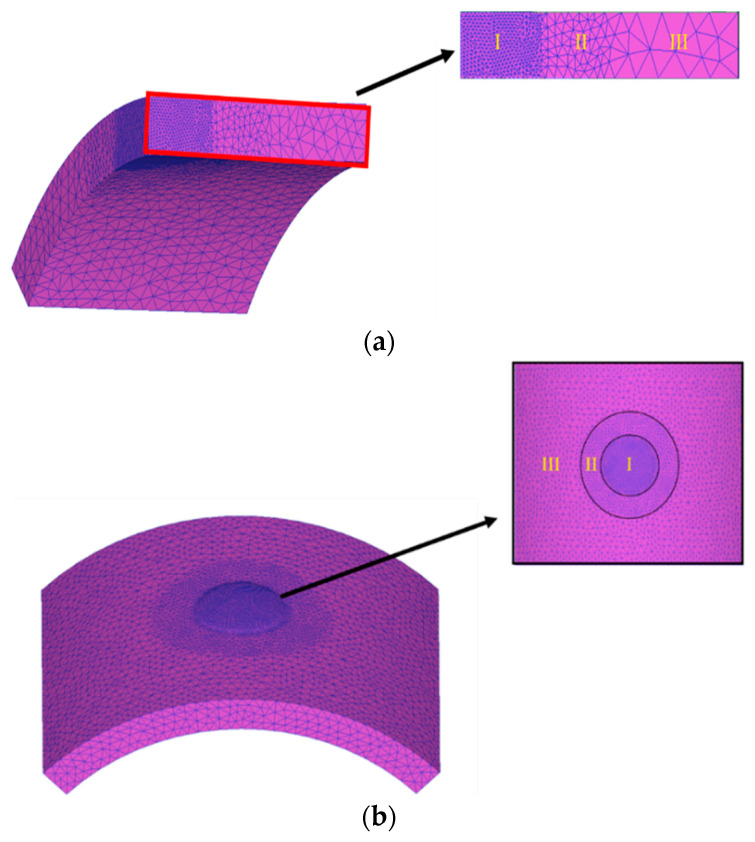
(**a**) Mesh division of the quarter model. (**b**) Mesh division of the full 3D model.

An implicit and static FE code, DEFORM 3D (V11.0.2) was adopted to analyze the elasto-plastic deformation pattern of an aluminum alloy tube during hydro-joining processes. The finite element code is based on the flow formulation approach using an updated Lagrange procedure. At first, the geometries of the objects are constructed using commercial software Solidworks (2023). Then, DEFORM 3D is used to implement the simulation of hydro-joining processes. The punch and die are regarded as rigid bodies, and the tube is elasto-plastic. The flow stresses of the aluminum alloy A6063 at different strain rates are shown in Figure 8, which were obtained by tensile tests. Due to symmetry on the left-right and front-back sides, only one quarter of the objects was adopted in simulations to save simulation time. One quarter of the tube, having a total of about 50,000 tetrahedral elements is divided into three zones, which have different element size ratios, set as 0.2, 0.4, and 1 for zones I, II, and III, respectively, as shown in Figure 9a. The tube material in zone I just below the punch head will undergo the severest plastic deformation; thus, a smaller element size ratio was set in this region. It is known that there are over ten layers of meshes in zones I and II in the thickness direction of the tube. The normalized Cockroft-Latham fracture criterion given in Table 2 was used to predict if the tube is pierced through by the punch or not in the hydro-joining processes. The critical damage value for A6063 was set as 1.42 [9].

The material parameters for the simulations are listed in Table 2, with Young’s modulus (E) set at 68.9 GPa and Poisson’s ratio (υ) set at 0.33. Both values were sourced from the DEFORM software database. The material was assumed to be homogeneous and isotropic. The failure criterion applied was that suggested by the normalized Cockroft-Latham theory, with the critical failure (Cf) set at 1.42 following the precedent of the study of Wu et al. [9]. Simulations were conducted at room temperature. For the contact interfaces, the Coulomb friction coefficient (μ) between the nut and the tube was set at 0.15, and that between the die and the tube was set at 0.2. Various nut fit zone geometries were combined with different punch stroke lengths and internal pressures to investigate their effects on nut hydro-joining. According to the dimensions and yield stress of aluminum alloy A6063 tubes used in this paper, the minimal internal pressure that causes the plastic deformation of the tube is approximately 20 MPa. Accordingly, several pressure levels that are above the minimal critical pressure were selected to investigate the effects of the internal pressures on the formability of tube hydro-joining processes of A6063 tubes.

During the forming process, major deformation occurred in the forming region, necessitating mesh refinement. Mesh refinement enhanced the accuracy of analyses in high-deformation areas and enlarged the meshes outside these areas to substantially reduce the computational time required for the simulations. The meshes were divided into three regions with different element size ratios, as illustrated in Figure 9a,b: Regions I, II, and III were set to 0.2, 0.4, and 1 mm, respectively. After we defined the mesh refinement, the system automatically generated the meshes. The total number of meshes for the two model configurations are 110,000 and 60,000 meshes for the full 3D model and the quarter model, respectively.

**Figure 10:**
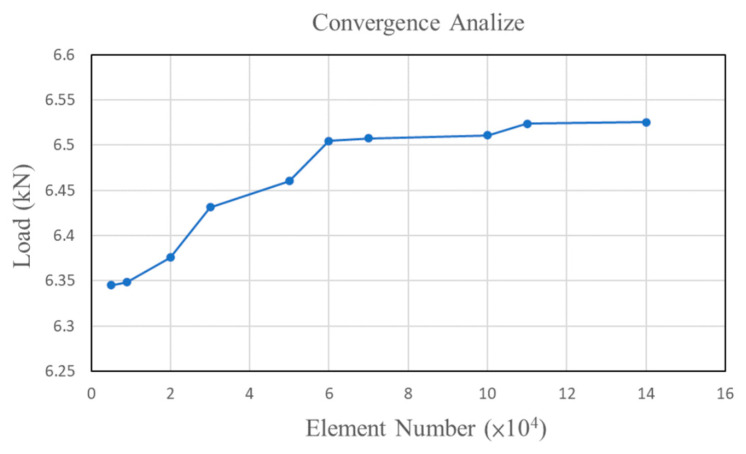
Effects of total element number on maximal punch forces.

Convergence analyses for simulation results were implemented to understand the ranges of the simulation result errors. Finite element simulations with different total element numbers were conducted. The effects of total element number on the maximal punch force during hydro-flanging processes are shown in Figure 10. Clearly, the relative differences in the maximal punch forces decrease to within 0.5% as the total element number increases to 60,000 elements. Accordingly, approximately 60,000 tetrahedron elements with a minimal element size of 0.2 mm were set for the tube object in the following finite element simulations.

## 3. Finite Element Simulation Results and Discussion

### 3.1. Forming Parameter Settings

**Figure 11:**
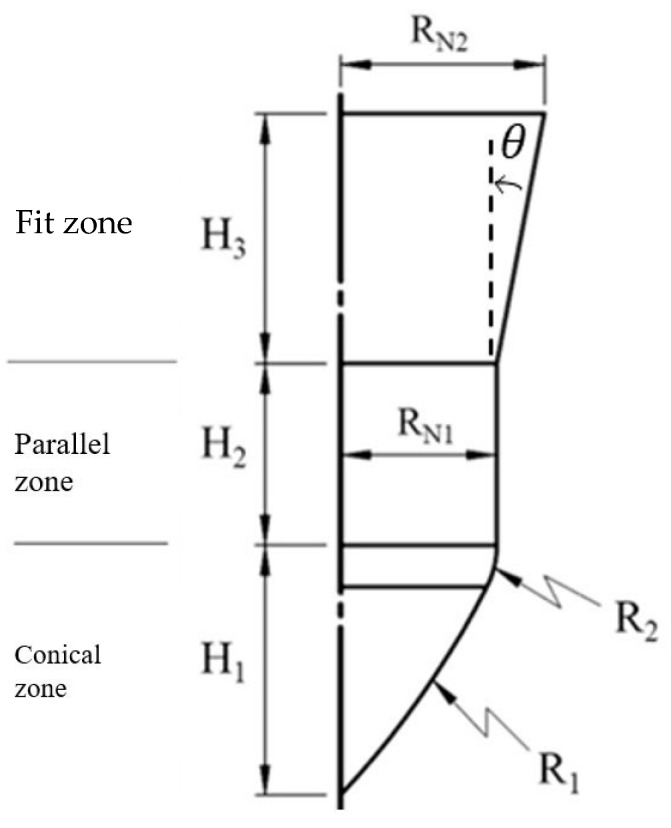
Reference diagram of nut parameter positions.

To investigate the effects of the geometric parameters of the nut fit zone on the pull out load during the hydro-joining of aluminum alloy A6063 tubes, simulations were conducted using various nut fit zone geometries, punch stroke lengths, and internal pressures. As indicated in Table 3 and Figure 11, six heights (H_3_) and four diameters (2R_N2_) were selected for the nut fit zone to explore the effects of tapered locking parts under different slopes.

### 3.2. Effects of Nut Fit Zone Parameter Changes on Pull Out Load

**Figure 12:**
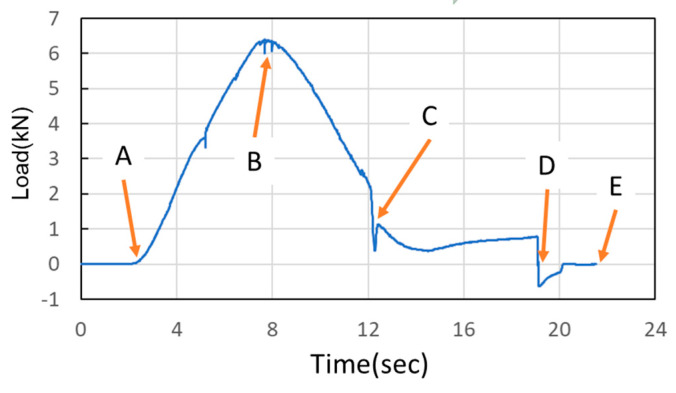
Nut load curve.

In the simulations, the load began at Point A, where the nut moves downward for hydro-piercing, causing the load to rise (Figure 12). At Point B, the load reached its peak, driven by internal pressure as the widest diameter of the conical zone passed through the tube. Between Points B and C, the nut continued to move downward, creating a smooth burnished surface due to shear fracture. At Point C, the fit zone of the nut began to enter the tube. At this stage, the punch-shaped nut temporarily ceased its downward motion, and the release of internal pressure resulted in a sharp decline in the load. Once the nut passed Point C, it resumed its downward motion to begin inlaying. With no internal pressure applied, the locking part design (secondary hole expansion) induced a slight load increase. Finally, at Point D, the punch began to withdraw, and by Point E, the load had reversed and was negative. In the hydro-piercing and hydro-flanging processes, the punch moves downward, and the load is shown with positive values. Whereas, in the pull out process, the punch moves in the opposite direction; accordingly, the load is shown with negative values.

Table 4 lists the associations between the fit zone height (H_3_) and punch stroke length. When the nut diameter (2R_N2_) is fixed, changes in the fit zone height (H_3_) affect the slope of the fit zone, enabling the effect of fit zone slope on punch stroke length to be calculated. During the inlaying stage, the top of the punch-shaped nut was inlaid aligned with the tube surface to ensure the sheet was properly formed. Therefore, the punch strokes were designed to correspond to the varying nut lengths resulting from the use of different fit zone heights.

**Figure 13:**
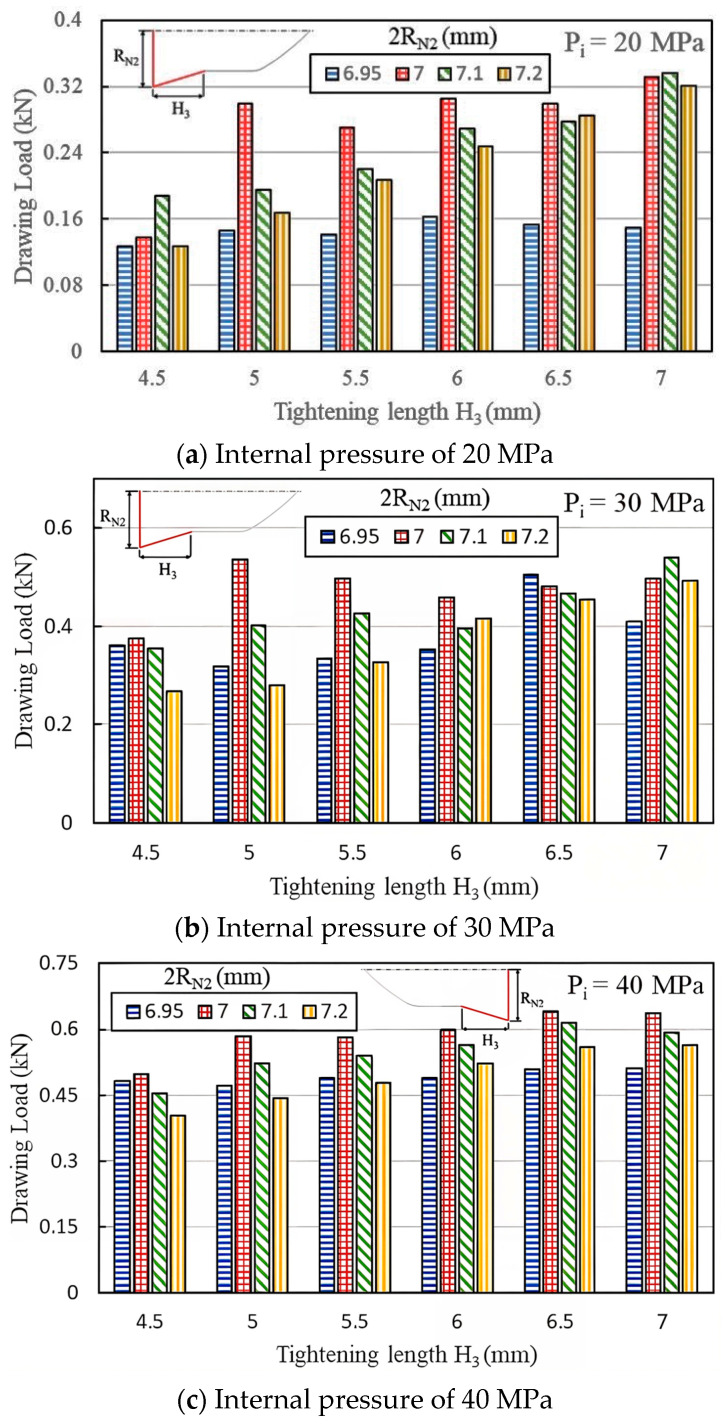

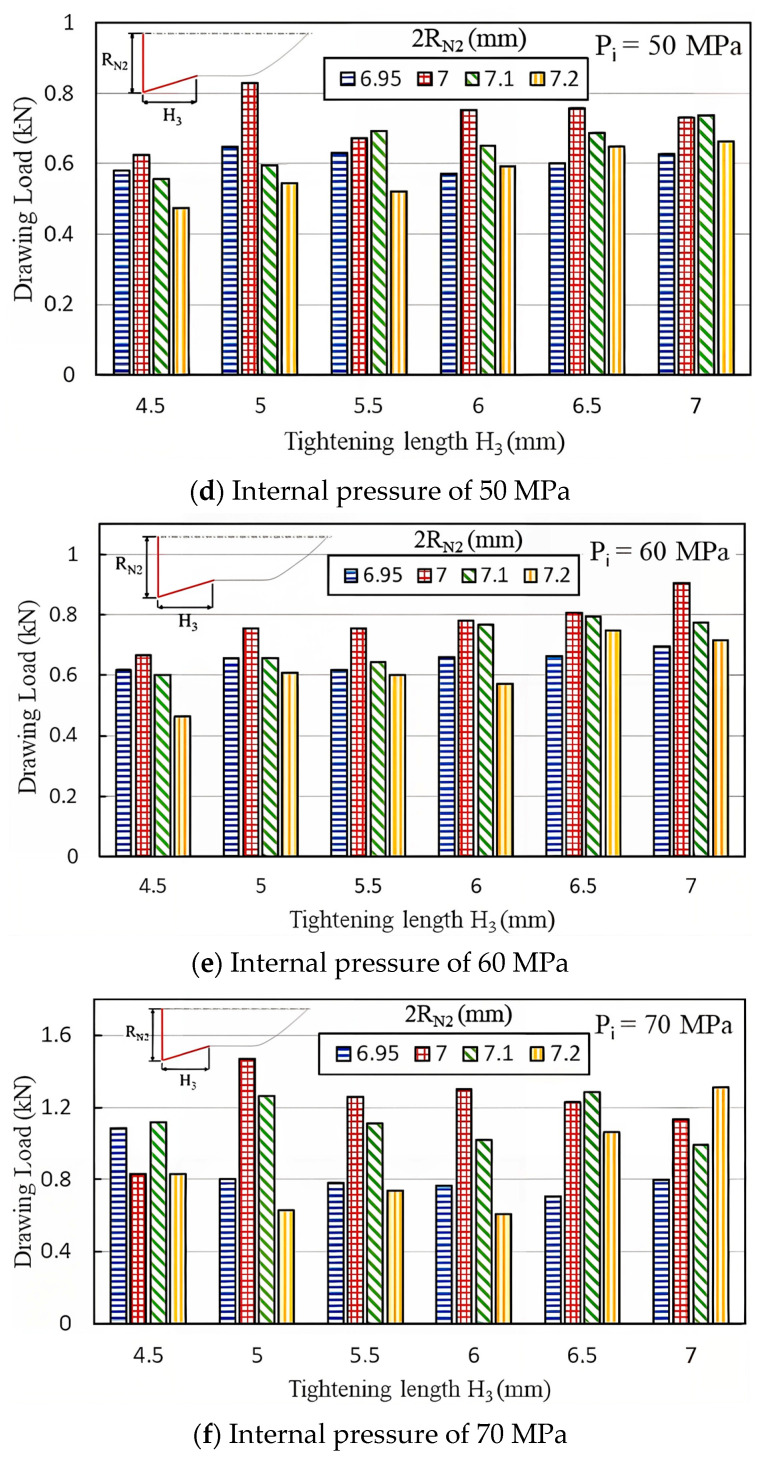
Maximum pull out load at various internal pressures.

Figure 13a–f compare the maximum pull out load under fit zone diameters (2R_N2_) of 7, 7.1, and 7.2 mm, six fit zone heights (H_3_), and six internal pressure levels. At internal pressures of 40, 50, and 60 MPa, the load decreased as the diameter increased from 7 mm to 7.2 mm. This result suggests that as the fit zone diameter (2R_N2_) increased, the fit zone slope also increased, causing material around the inlaying hole to undergo plastic deformation and reducing the clamping force. Similar trends were observed at 20 and 30 MPa. However, at 70 MPa, the load became unstable due to material overflow. Therefore, a fit zone diameter of 7 mm achieved a superior pull out load to diameters of 6.95, 7.1, and 7.2 mm, indicating that extremely small or large fit zones did not enhance the clamping force.

Figure 13a–f show the effects of the tightening length H_3_ at the fit zone on the pull out load at internal pressures of 20, 30, 40, 50, 60, and 70 MPa, respectively. Different internal pressures result in different geometric configurations between the punch and pierced tube in the hydro-joining process. Accordingly, the net diameter 2R_N2_ and the tightening length H_3_ exhibit different effects on the pull out loads.

### 3.3. Torque Analyses and Discussion

**Figure 14:**
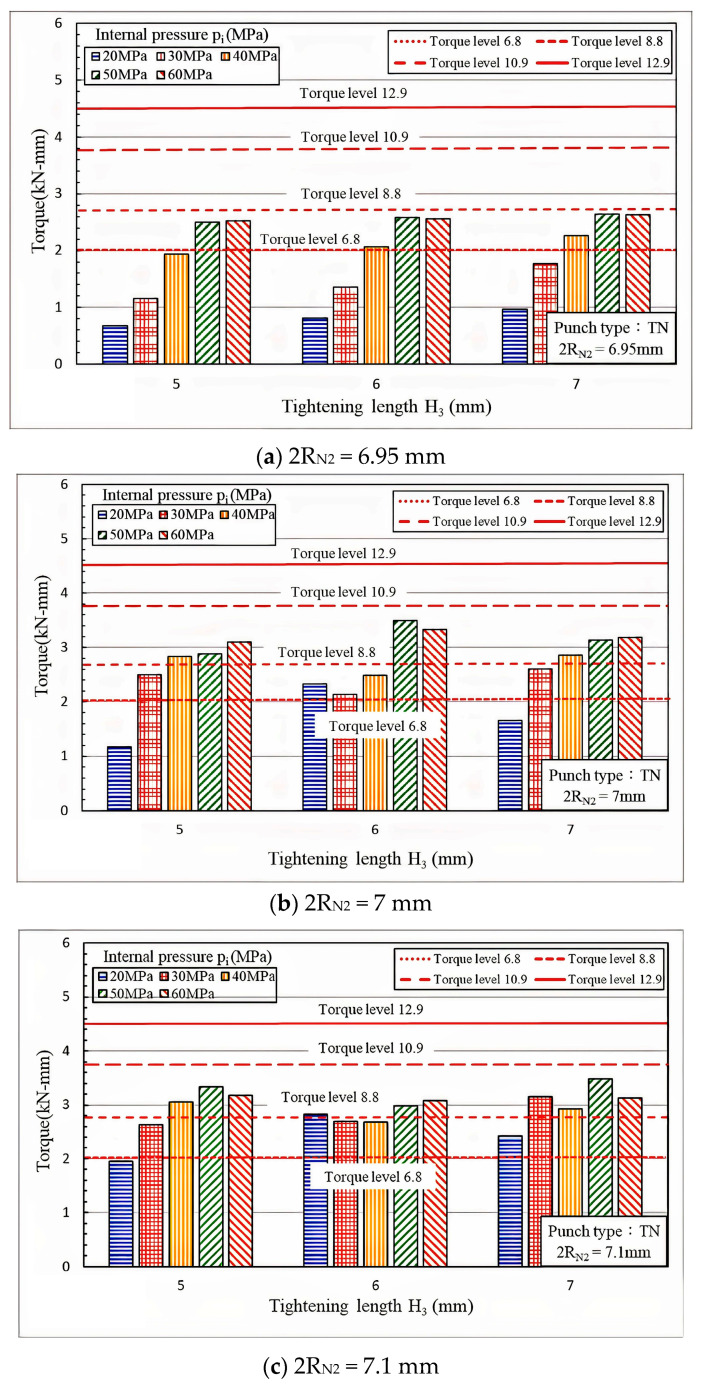
Torque comparison at various fit zone diameters (2 R_N2_).

Table 5 presents the torque simulation parameters. In the torque simulations, the internal pressure was set to levels that would prevent material overflow, which could render demolding difficult. To simplify the simulation groups, the fit zone heights (H_3_) were limited to 5, 6, or 7 mm. On the basis of findings indicating that a 2R_N2_ of 7 mm exhibited the optimal pull out load performance and the clearest trends, this study focused on a 2R_N2_ of 6.95 and 7.1 mm in the torque analyses. Because no standards have been established for nut torque, the torque values were compared with industry standards for bolts. The bolt performance grades assessed were 6.8, 8.8, 10.9, or 12.9, with the digits in the tens and ones places representing the tensile strength and yield ratio, respectively. For example, a bolt with a grade of 4.8 indicates a material tensile strength of 400 MPa and a yield ratio of 0.8, resulting in a yield strength of 400 × 0.8 = 320 MPa. As illustrated in Figure 14a–c, the nut torque values for the nuts in the present study were all below grade 10.9 and thus did not meet the standards for pneumatic tool operations.

In addition to pull out load, twisting torque is also an important evaluation factor in tube hydro-joining processes. From these figures, we can realize under what conditions the maximal twisting torque generated between the nut and tube can meet the required torque levels.

**Figure 15:**
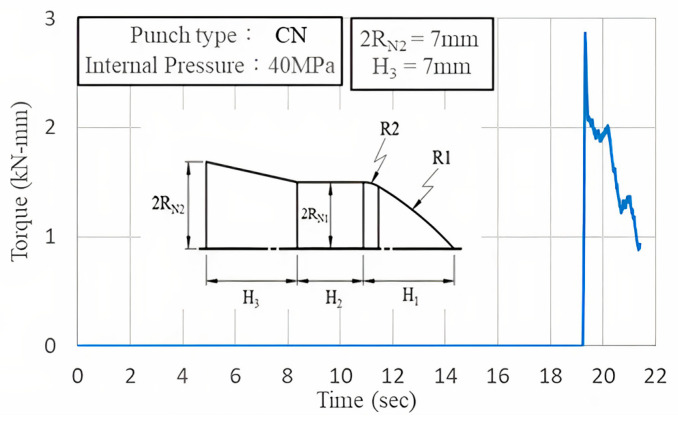
Nut torque curve.

Figure 15 illustrates the torque curve of the nut during the first stage of punching under an internal pressure of 40 MPa. The nut torque reached a peak at the initial stage of rotation and subsequently decreased as the geometric shape eliminated interference.

## 4. Nut Hydro-Joining Experiments

### 4.1. Experimental Die Design and Equipment Overview

**Figure 16:**
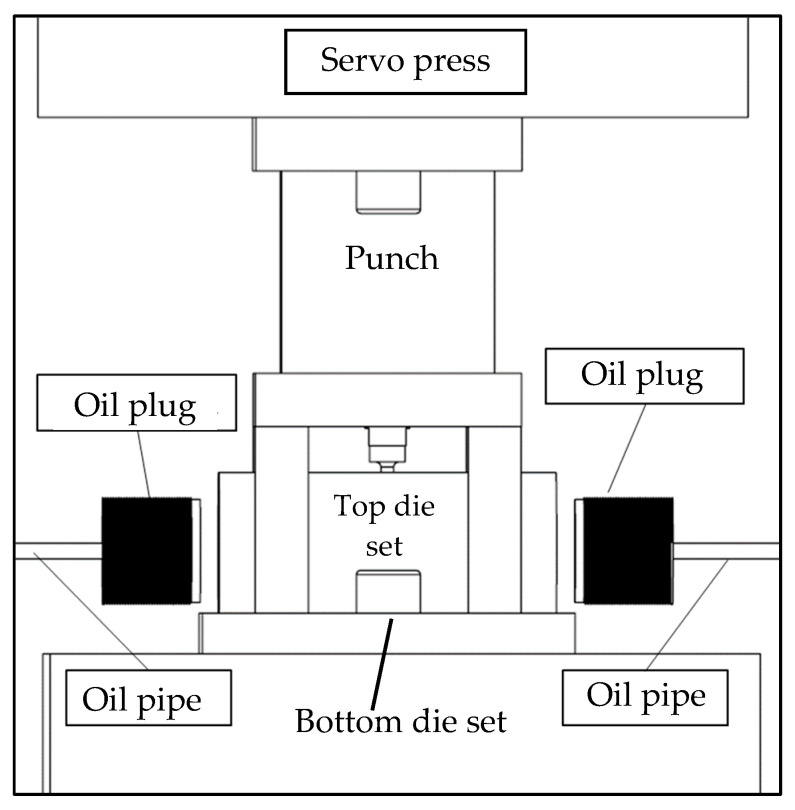
Experimental equipment configuration.

Figure 16 illustrates the configuration of the experimental equipment. Before the experiment, the tube was placed into the tube-holding sleeve of the lower die set. The external hydraulic motor delivered hydraulic fluid through oil pipes and plugs into the tube-holding sleeve of the lower die set. The upper and lower die sets were fixed to a punch machine for punching.

**Figure 17:**
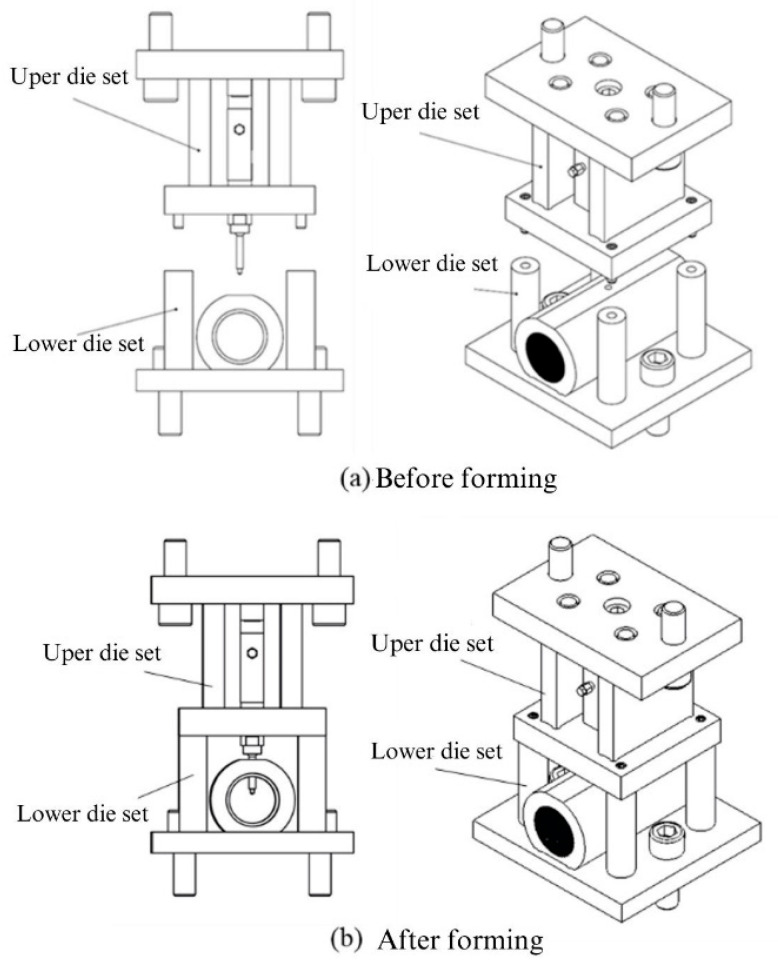
Hydro-piercing and flanging die set assembly diagram.

For the experiments in this study, custom-designed upper and lower die sets were employed to integrate the hydraulic motor, servo press, load cell, and tube-holding sleeve. The die set assembly is depicted in Figure 17. The upper die set served as the punch mechanism driving the nut during the punching process. The lower die set was used to secure the tube, connect to the hydraulic tubes, and position and support the upper die set during assembly. Figure 17a illustrates the die set assembly before the forming stage. At this stage, a tube can be placed into the tube-holding sleeve without geometric interference because the punch is retracted. Figure 17b depicts the die set assembly after the forming stage, in which the upper and lower die sets are aligned and positioned using guide pins fixed to the servo press for the experiments.

**Figure 18:**
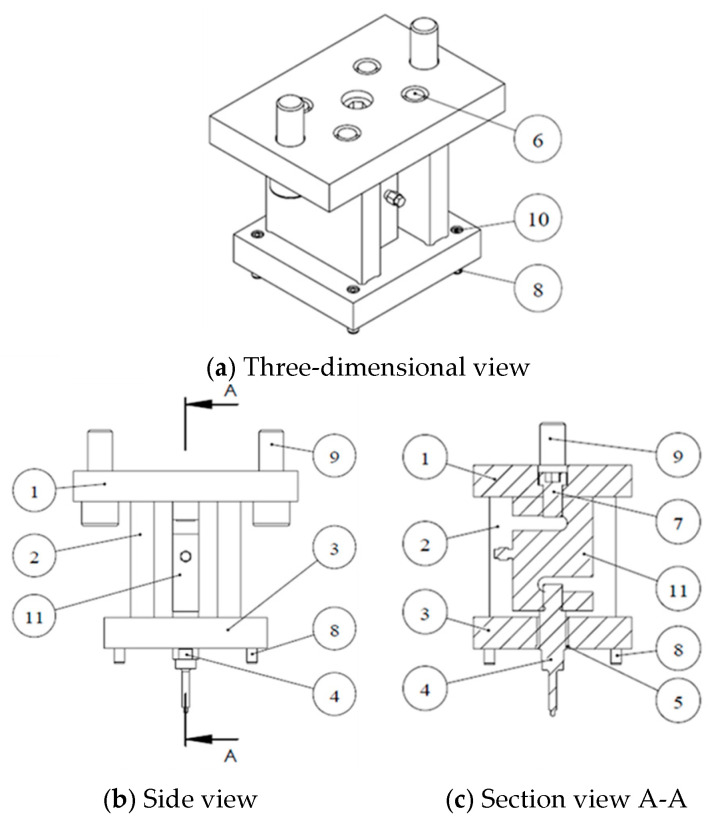
Upper die set assembly diagram.

As indicated in Figure 18 and the parts list in Table 6, the upper die set was primarily designed to connect the punch and load cell to the servo press. A load-cell-fixing die was designed to ensure precise alignment between the punch and the die hole on the tube-holding sleeve in the lower die during die set assembly. The load-cell-fixing die also supported the upper die plate and prevented the load cell from being subjected to preload when the upper and lower die sets were fixed to the upper guide pillar of the servo press. The guide sleeve and guide pins ensured that the punch and die hole were accurately aligned. The load cell used in this experiment was a commercially available ST1-2.5T model, with a measurement range from 5 kg to 2.5 tons. This load cell was selected on the basis of FE simulation results. Before the experiments, the load cell was calibrated to ensure the accuracy of the measurements.

**Figure 19:**
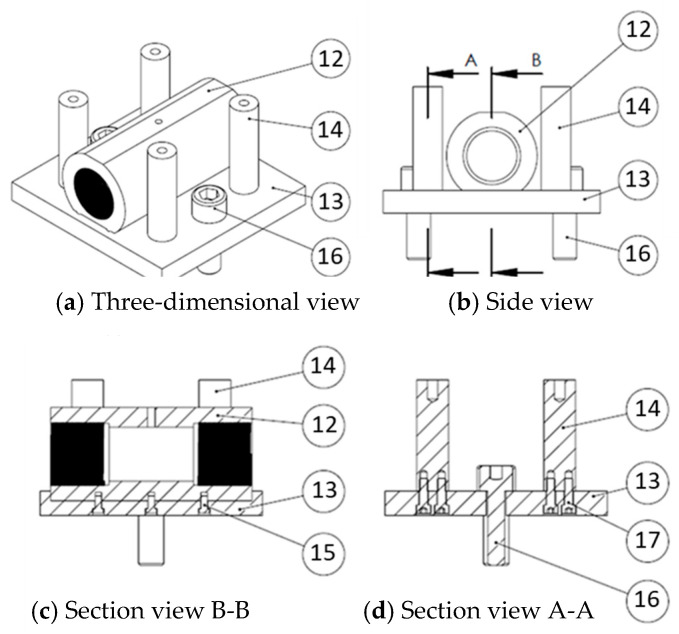
Lower die set assembly diagram.

As Figure 19 and the parts list in Table 7 demonstrate, the lower die set was primarily designed to connect the tube-holding sleeve to the servo press. The guide pillars were used to ensure precise alignment during die set assembly and to support the upper die set when the two die sets were fixed to the servo press, facilitating assembly. Holes were drilled in the top of the guide pillars to align with the upper die set guide pins, ensuring accurate positioning of the die and precise alignment of the punch and die hole.

**Figure 20:**
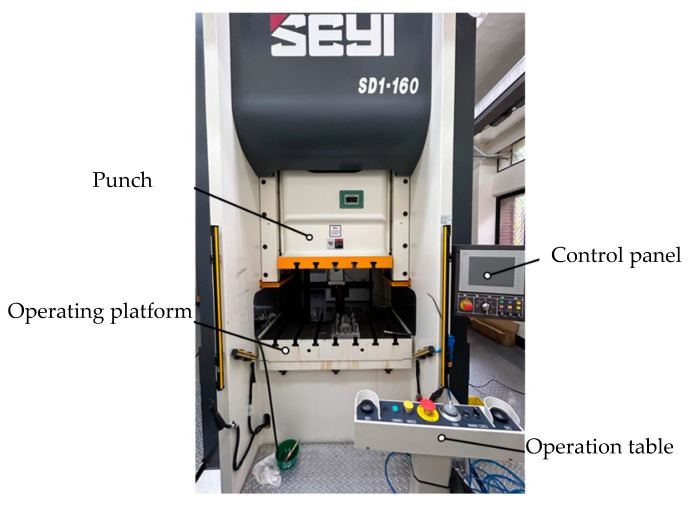
Servo press SD1-160.

A servo press (model SD1-160) manufactured by Shieh Yih Machinery Industrial (Figure 20) with a capacity of 160 tons and a maximal punch stroke of 220 mm was used in this experiment. The experimental parameters, including die height, punch stroke length, dwell speed, and punching speed, were inputted through the control panel, with the machine operations controlled using the operating platform. The hydraulic pressure required was provided by an external, self-designed hydraulic system. The experimental load was measured using the load cell installed in the upper die set. Data were captured using a data acquisition system and transferred to a computer for analyses.

### 4.2. Experimental Procedure and Design

In the experiments of aluminum alloy hydro-joining processes, three physical quantities were measured. They were punch loads, internal pressures, and punch displacements or strokes. A load cell that can measure tensile and compressive forces was installed between the upper die plate and the nut punch, as shown in Figure 18 and Table 6. The accuracy of the load cell is ±10 kg. Internal pressure was measured by a pressure transducer and indicated by a pressure gauge with an accuracy of ±0.1 MPa. The punch stroke is recorded by the servo press automatically during the experiments. Its accuracy is ±0.02 mm. Three repetitions were performed for each experiment, and the maximal deviation for the load variations is about 5%.

**Figure 21:**
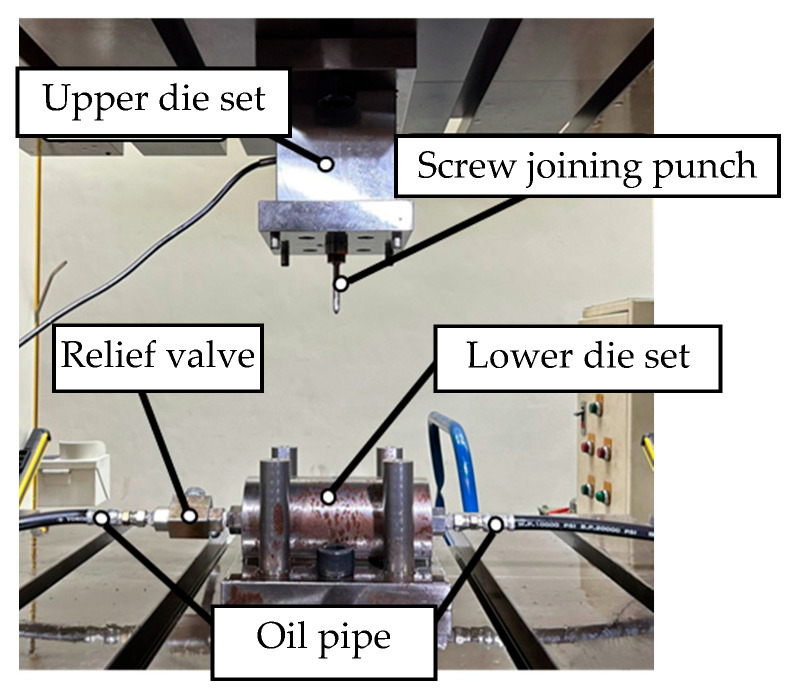
Completed die set assembly.

On the basis of the simulation results, hydro-joining experiments were conducted with a fit zone height (H_3_) of 7 mm and a nut diameter (2R_N2_) of 7 mm under hydraulic pressures of 30 MPa and 50 MPa. The tube material used was annealed aluminum alloy A6063, with a length, diameter, and thickness of 83, 50.8, and 3 mm, respectively, to fit the die dimensions. The pull out load results from the experiments were compared with the simulation results. Prior to the experiment, the tubes underwent annealing. The annealing process involved maintaining the tubes at a temperature of 420 °C for 3 h, followed by cooling at a rate of 30 °C per hour to 260 °C, and finally natural cooling to room temperature. The upper and lower die sets were fixed to the punch and the servo press operation table. The punch was retracted from the lower die set using the servo press, after which the tube was placed into the tube-holding sleeve, and an oil plug was installed. Subsequently, the hydro-joining experiment was conducted. The nuts were attached or replaced using the threads on the nut-joining punch. Figure 21 depicts the completed die set assembly used in the experiments.

### 4.3. Pull out Load Experiment Results

**Figure 22:**
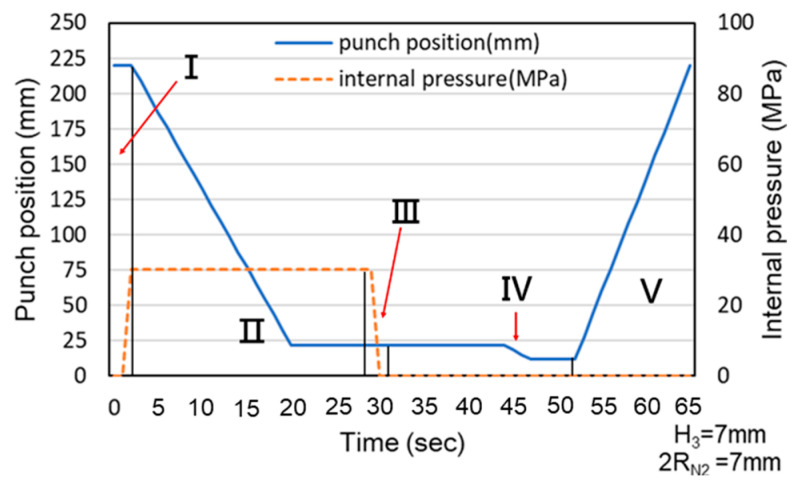
Association between punch stroke length and an internal pressure of 30 MPa.

**Figure 23:**
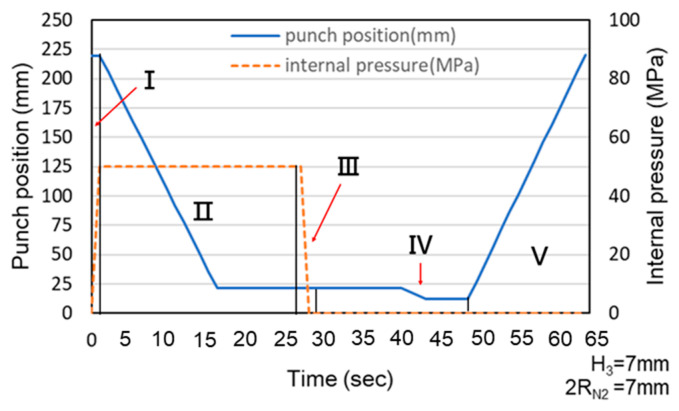
Association between punch stroke length and an internal pressure of 50 MPa.

Because the servo press was manually operated during the experiments, the load curve exhibited delays caused by human activity. These delays explain the observed association between punch stroke and internal pressure and the stages represented by each section of the experimental load curve. Additionally, the experimental and simulated results were compared using the maximum load and maximum pull out load. Figure 22 and Figure 23 illustrate the relationship between punch stroke length and internal pressure during the experiments. The physical meaning of region numbers I–V is the same as that in Figure 6. The curve in these figures indicates that the experimental procedure was consistent with the procedure in the simulation.

**Figure 24:**
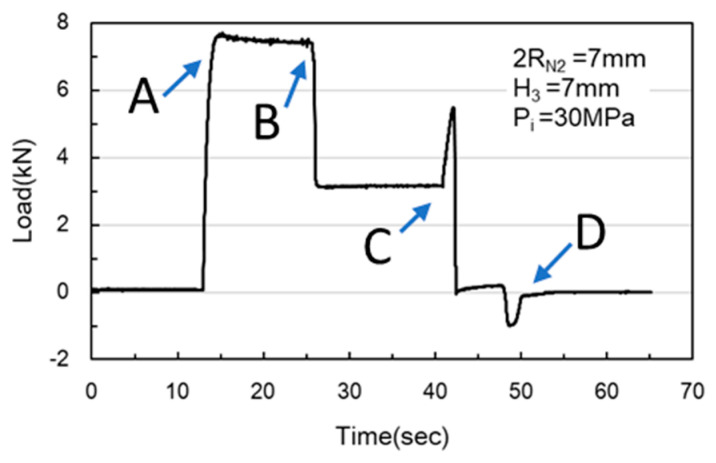
Load curve measured at an internal pressure of 30 MPa.

**Figure 25:**
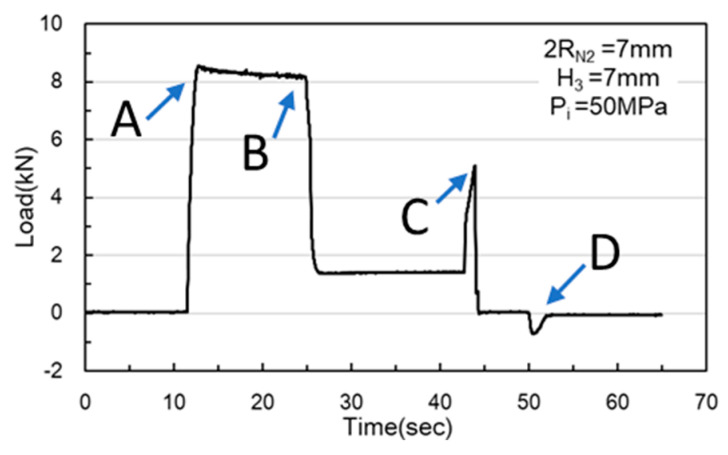
Load curve measured at an internal pressure of 50 MPa.

As Figure 24 and Figure 25 indicate, Point A represents the maximum load induced by the downward movement of the nut and the upward hydraulic force during hydro-joining. Point B represents the load reduction after the hydraulic pressure is released. Point C represents the nut repunching stage during inlaying. Point D corresponds to the maximum pull out load region during nut removal.

**Figure 26:**
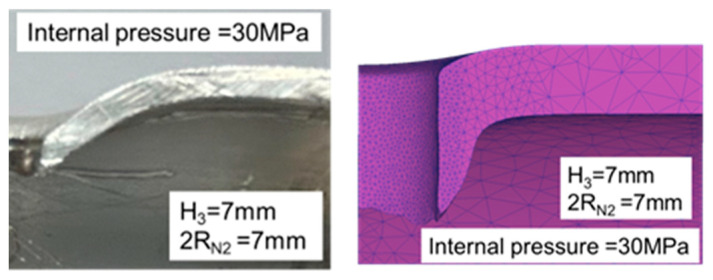
Comparison of the appearance of the cross-section between the experiments and simulations at an internal pressure of 30 MPa.

**Figure 27:**
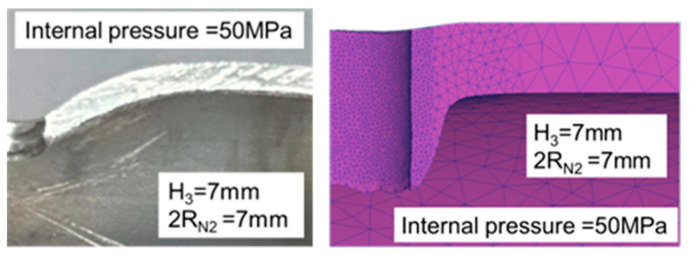
Comparison of the appearance of the cross-section between the experiments and simulations at an internal pressure of 50 MPa.

As the results presented in Table 8 demonstrate, the error in the maximum pull out load results was approximately 20%, indicating that the experimental results were consistent with those of the simulations. As the results presented in Table 9 indicate, the error in the maximum pull out load was larger at 30 MPa, approaching 50%, whereas the error at 50 MPa was only 5%. As depicted in Figure 26 and Figure 27, the punch edge length in the simulations was longer than that in the experiments because in the simulations, the material flowed and ruptured at the nut’s tip before being pushed outward by the R20 rounded corner of the nut, forming a flange. In the experiments, the rupture zone clearly appeared near the nut’s parallel zone, resulting in a shorter flange length and the production of waste material. The rollover in the experiments was also significantly larger, possibly due to the lack of internal pressure support after the pressure release during the nut inlaying stage, which led to excessive punch strokes. Additionally, the failure zone appeared in the annular region rather than the tip region as in the simulations, which also contributed to the larger error in the experiments.

### 4.4. Nut Inlaying Experiment

**Figure 28:**
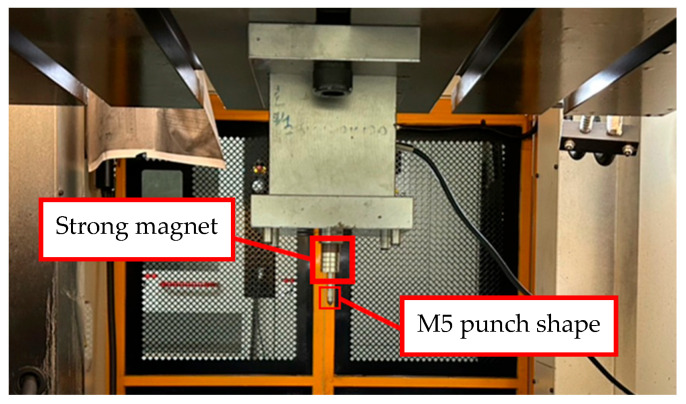
Configuration of experimental inlaid aluminum and nut in the nut inlaying experiments.

**Figure 29:**
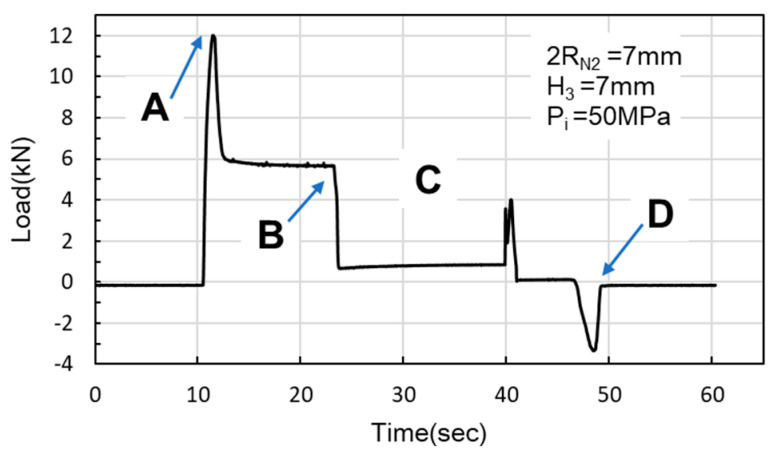
Load curve of nut inlaying experiment.

M5 threads larger than the original M4 threads inside the nuts were designed to enable nut inlaying (Figure 28). The purpose of these threads was to prevent the nuts from being pulled out along with the punch during the pull out process. To achieve this, a powerful annular magnet was added above the nuts to prevent separation of the nut and punch due to gravity. The nut inlaying experiment used nonannealed A6063 tubes, which resulted in harder material and less rollover. Figure 29 depicts the load curve for the nut inlaying experiment. Point A represents the maximum load induced by the downward movement of the nut and the upward hydraulic force during the hydro-joining of the nut. Point B represents the load reduction after the hydraulic pressure was released. Point C represents the nut repunching stage during inlaying. Point D is the point at which the punch begins to pull out; at this point, the nut is embedded into the tube, making the load value 0.

**Figure 30:**
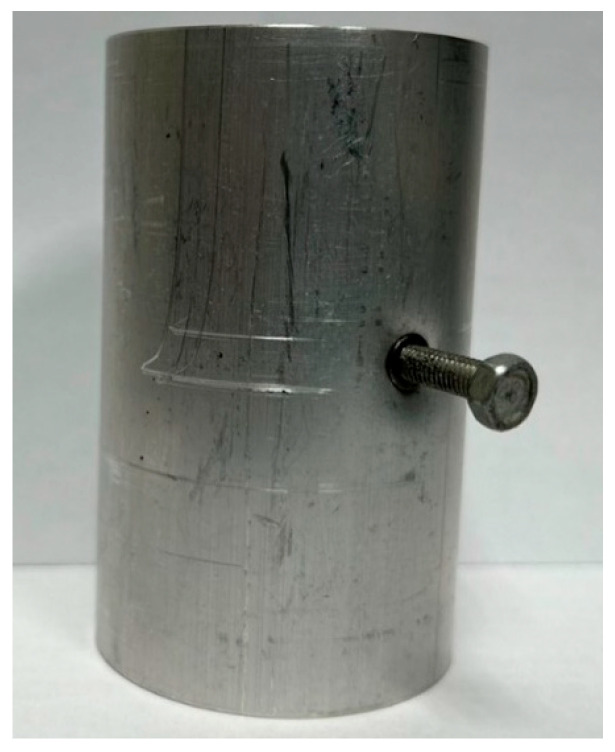
Appearance of tube and bolt connection after hydro-joining.

Table 10 presents the appearance of the nut with M5 threads at 2R_N2_ = 7 mm and H_3_ = 7 mm. Inlays at all three parameter values were successful and locked bolts, as illustrated in Figure 30. Although the 0 MPa condition resulted in a successful nut inlay, a noticeable rollover was present on the surface. At 30 MPa, a slight, nearly invisible rollover was present. At 50 MPa, the surface was completely flat, indicating optimal formability. In the images in the figure at pressures of 30 MPa and 50 MPa, waste material from hydropiercing visibly adheres to the nuts’ conical zones, preventing waste material from entering the hydraulic recovery system. Table 10 shows the appearance of the rollover around the pierced hole at different internal pressures. A smaller rollover is beneficial for a larger pull out load. Accordingly, a larger pull out load was obtained at 50 MPa than at 30 MPa.

## 5. Conclusions

This study conducted a series of analyses and experiments on tube hydro-joining of aluminum alloy A6063. From the comparisons of the maximal punch loads between the simulations and experiments, errors of 24% and 19% for internal pressures of 30 MPa and 50 MPa, respectively, were found. Some conclusions are listed below.

(1) The pull out load steadily increased as the internal pressure increased, but at an internal pressure of 70 MPa, the material overflowed into the die cavity, affecting the pull out load simulations.
(2) The load slightly increased as the fit zone height increased, likely due to the increased punch stroke length caused by the fit zone height changes, which increased the contact area between the nuts and the tubes, increasing the pull out load. By contrast, as the fit zone diameter decreased from 7.2 mm to 7 mm, the pull out load increased. The pull out load increased until the fit zone diameter reached 6.95 mm; subsequently, it began to decrease. Therefore, this study concluded that the optimal parameters for a large load are a fit zone diameter of 7 mm and an internal pressure of 60 MPa, which result in a 906 N pull out load.
(3) The nut torque simulation results revealed that due to nut geometry, the rotation was smooth, and the torque typically was below or between torque grades 8.8 and 10.9, failing to meet the standards for pneumatic tools and professional operations.
(4) In the hydro-joining experiment results, the error in the maximum pull out load was relatively large at 30 MPa, approaching 50%, whereas the error at 50 MPa was only 5%. Substantial rollover was observed, likely due to the lack of internal pressure support during the nut inlaying stage after pressure release, which led to excessive punch strokes. This study also observed that the failure zone in the experiments occurred in the annular region rather than at the tip region as in the simulations, contributing to the larger observed error in the experiments.

In the future, the tube materials for hydro-joining can be changed to carbon steels that have higher stiffness and are beneficial for raising the pull out strength. The internal pressure needed for tube hydro-joining of carbon steels is probably above 100 MPa; thus, a pressure intensifier needs to be designed to raise the hydraulic pressure. Concerning the applications of tube hydro-joining, carbon steel or aluminum alloy tubes used in engine cradles or frame rails of automobiles are usually hydroformed into desired tube shapes firstly, and then tube hydro-joining processes are applied to join two separate parts in the engine cradles or frame rails.

## Figures and Tables

**Table 1:** Parameter design of the punch-shaped nut.

Nut Diameter 2R_N_ [mm]	6.9
Nut height H_3_ + H_2_ [mm]	4
Nut height H_1_ [mm]	4
Nut height H_N_ [mm]	5.5
Position of Center R_1_	
X_10_ [mm]	−14.58
Z_10_ [mm]	13.69
Position of Center R_2_	
X_20_ [mm]	1.45
Z_20_ [mm]	5.5

**Table 2:** Simulation parameter settings.

FEM Software	DEFORM 3D	
flow stress	$\bar{\sigma }=\bar{\sigma }\left(\bar{\varepsilon },\dot{\bar{\varepsilon }}\right)$	
Failure criteria	Normalized C and L: $C_{f}=\int_{0}^{{\bar{\varepsilon }}_{f}}\frac{\sigma_{1}}{\bar{\sigma }}d\bar{\varepsilon }$	
Critical value C_f_	1.42	
Young’s modulus E [GPa]	68.9	
Poisson’s ratio υ	0.33	
Coefficient of friction μ	Nut and Tube	0.15
	Die and Tube	0.2
Punch velocity V [mm/s]	1	
Internal pressure p_i_ [MPa]	20, 30, 40, 50, 60, 70	
Temperature T	Room temperature	

**Table 3:** Simulation parameters for nut pull out load analyses.

Simulation Type	Quarter Model
Internal pressure, P_i_ [MPa]	20, 30, 40, 50, 60, 65, 70
Die hole diameter, 2R_h_ [mm]	7.2
Nut fit zone height, H_3_ [mm]	4.5, 5, 5.5, 6, 6.5, 7
Nut parallel zone height, H_2_ [mm]	4
Nut conical zone height, H_1_ [mm]	5.5
Nut diameter, 2R_N2_ [mm] (clearance value)	6.95, 7, 7.1, 7.2 (0.125, 0.1, 0.05, 0)
Nut diameter, 2R_N1_ [mm]	6.9
Rounded corner, R_2_ [mm]	2
Rounded corner, R_1_ [mm]	20

**Table 4:** Relationship between fit zone height (H_3_) and punch stroke.

Fit zone height, H_3_ [mm]	4.5	5	5.5	6	6.5	7
Punch stroke length [mm]	14	14.5	15	15.5	16	16.5

**Table 5:** Nut torque simulation parameters.

Simulation Type	Full 3D Model
Internal pressure, P_i_ [MPa]	20, 30, 40, 50, 60
Die hole diameter, 2R_h_ [mm]	7.2
Fit zone height, H_3_ [mm]	5, 6, 7
Parallel zone height, H_2_ [mm]	4
Conical zone height, H_1_ [mm]	5.5
Diameter, 2R_N2_ [mm] (clearance value)	6.95, 7, 7.1 (0.125, 0.1, 0.05)
Diameter, 2R_N1_ [mm]	6.9
Rounded corner, R_2_ [mm]	2
Rounded corner, R_1_ [mm]	20

**Table 6:** Upper die set part list.

No.	Name	Material	Quantity
1	Upper die plate	S45C	1
2	Load cell-fixing die	S45C	2
3	Load cell-fixing die	S45C	1
4	Nut punch	SKD11	1
5	Guide sleeve	SUJ2	1
6	M10×30 hexagon bolt	SCM435	8
7	M18×30 hexagon bolt	SCM435	1
8	Guide pin	SUJ2	4
9	M24×70 hexagon bolt	SCM435	2
10	M6×16 hexagon bolt	SCM435	4
11	Load cell		1

**Table 7:** Lower die set parts list.

No.	Name	Material	Quantity
12	Tube-holding sleeve	Recycled steel	1
13	Lower die plate	S45C	1
14	guide pillar	SS41	4
15	M6×12 hexagon bolt	SCM435	3
16	M24×70 hexagon bolt	SCM435	2
17	M8×25 hexagon bolt	SCM435	8

**Table 8:** Comparison of the maximum load between the simulations and experiments.

	Simulation [kN]	Experiment [kN]	Error Value
30 MPa	5.87	7.71	24%
50 MPa	6.94	8.56	19%

**Table 9:** Comparison of the maximum pull out load between the simulations and experiments.

	Simulation [kN]	Experiment [kN]	Error Value
30 MPa	0.51	1.004	49%
50 MPa	0.75	0.711	5%

**Table 10:** Appearance of hydro-joining nut and tubes under different pressures.

P_i_ = 0 MPa 2R_N2_ = 7 mm H_3_ = 7 mm	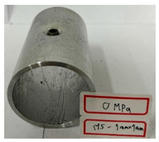 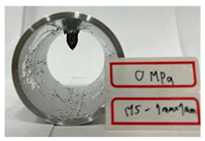
P_i_ = 30 MPa 2R_N2_ = 7 mm H_3_ = 7 mm	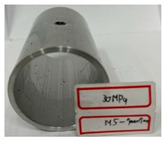 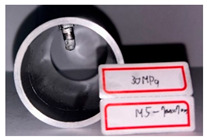
P_i_ = 50 MPa 2R_N2_ = 7 mm H_3_ = 7 mm	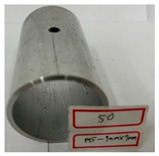 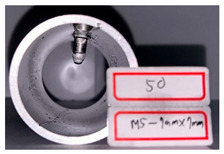

## Data Availability

The original contributions presented in this study are included in the article. Further inquiries can be directed to the corresponding author.

## Nomenclature

2R_h_	Die hole diameter
P_i_	Internal pressure
P_p_	Punch position
T	Time
R_N1_	Top of fit zone radius
R_N2_	Fit zone radius
H_3_	Fit zone height
H_2_	Parallel zone height
H_1_	Conical zone height
R_1_	Profile radius of conical zone
R_2_	Corner radius
